# Treatment of the bone marrow stromal stem cell supernatant by nasal administration—a new approach to EAE therapy

**DOI:** 10.1186/s13287-019-1423-6

**Published:** 2019-11-15

**Authors:** Xi Wang, Wantong Zhai, Jiahui Zhu, Wei Zhao, Xiaoyi Zou, Siying Qu, Shenyue Wang, Zhongze He, Zhaoying Li, Lingyang Wang, Bo Sun, Hulun Li

**Affiliations:** 1 Department of Neurobiology, Harbin Medical University, Harbin, 150086 Heilongjiang China; 2 The Key Laboratory of Myocardial Ischemia, Harbin Medical University, Ministry of Education, Harbin, 150086 Heilongjiang China; 3 Department of Cardiology, The Second Affiliated Hospital of Harbin Medical University, Harbin, 150086 Heilongjiang China

**Keywords:** BMSCs, Nasal administration, EAE, B cells

## Abstract

**Introduction:**

Multiple sclerosis (MS) is one of the most common autoimmune diseases of the central nervous system (CNS). CNS has its own unique structural and functional features, while the lack of precision regulatory element with high specificity as therapeutic targets makes the development of disease treatment in the bottleneck. Recently, the immunomodulation and neuroprotection capabilities of bone marrow stromal stem cells (BMSCs) were shown in experimental autoimmune encephalomyelitis (EAE). However, the administration route and the safety evaluation limit the application of BMSC. In this study, we investigated the therapeutic effect of BMSC supernatant by nasal administration.

**Methods:**

In the basis of the establishment of the EAE model, the BMSC supernatant were treated by nasal administration. The clinical score and weight were used to determine the therapeutic effect. The demyelination of the spinal cord was detected by LFB staining. ELISA was used to detect the expression of inflammatory factors in serum of peripheral blood. Flow cytometry was performed to detect pro-inflammatory cells in the spleen and draining lymph nodes.

**Results:**

BMSC supernatant by nasal administration can alleviate B cell-mediated clinical symptoms of EAE, decrease the degree of demyelination, and reduce the inflammatory cells infiltrated into the central nervous system; lessen the antibody titer in peripheral bloods; and significantly lower the expression of inflammatory factors. As a new, non-invasive treatment, there are no differences in the therapeutic effects between BMSC supernatant treated by nasal route and the conventional applications, i.e. intraperitoneal or intravenous injection.

**Conclusions:**

BMSC supernatant administered via the nasal cavity provide new sights and new ways for the EAE therapy.

## Introduction

Multiple sclerosis is a common debilitating disease of the central nervous system that is long believed to be of an autoimmune origin [1]. Typical syndromes at presentation include, but are not limited to, monocular visual loss due to optic neuritis, limb weakness or sensory loss due to transverse myelitis, double vision due to brain-stem dysfunction, or ataxia due to a cerebellar lesion [2, 3]. Compartmentalized inflammation within the CNS, including diffuse activation of innate myeloid cells, characterizes the progressive phase of MS, the most debilitating phase that currently lacks satisfactory treatments [4]. The blood-brain barrier also acts as a barrier that inhibits the delivery of some therapeutic agents to the CNS and hinders drugs from passing through the endothelial capillaries to the brain [5].

Bone marrow stromal cells, a type of multipotent stem cell [6], have the ability to adopt the fate of mesodermal, endodermal, and ectodermal cell types [7]. The ability for multilineage differentiation of BMSCs renders them potentially useful in treating various diseases [8], for example, acute myeloid leukemia [9, 10], aplastic anemia [11, 12], erectile dysfunction [13, 14], cirrhosis [15], eye diseases [16, 17], etc. Indeed, experimental studies in animals have reported that BMSCs also can ameliorate neurologic deficits and facilitate functional recovery in many disorders of the central nervous system, such as Parkinson’s disease [18], traumatic brain injury [19], spinal cord injury [20, 21], multiple sclerosis [22], and cerebral ischemia [23]. However, there are many problems with BMSC cell therapy, for example, it is easy to form iatrogenic tumor [24], has high differentiation risk, and cannot be controlled [25]. In contrast, the above problems are not present in the BMSC supernatant. Many studies focused on BMSCs’ ability to secrete a series of bioactive molecules, as cytokines and growth factors in response to diseases [26–28].

As a non-invasive treatment, intranasal administration bypasses the BBB and allows direct access to the brain through olfactory and trigeminal nerve pathways, which has led to its receiving significant attention in recent years [29, 30]. It offers advantages such as brain targeting, no gastrointestinal irritation, fast onset of action, avoidance of first-pass metabolism, and fewer systemic side effects [31]. In this study, we used the method of intranasal administration of BMSC supernatant to investigate the therapeutic effect of BMSC supernatant on experimental autoimmune encephalomyelitis (EAE), the animal model of MS. We found that BMSC supernatant have significant therapeutic effects on EAE, reducing the inflammatory infiltration and demyelination of the central nervous system and reducing the secretion of inflammatory cytokines in peripheral blood. These BMSC supernatant directly affect B cell, thereby changing the subtype of T cells.

EAE is the most commonly used experimental model for multiple sclerosis (MS). EAE is a complex condition in which the interaction between a variety of immunopathological and neuropathological mechanisms [32] leads to an approximation of the key pathological features of MS: inflammation [33], demyelination [34], axonal loss, and gliosis. The most common EAE model is induced by myelin oligodendrocyte glycoprotein peptide 35–55 (MOG_35–55_) [35, 36]; however, B cells are not effectively activated in MOG_35–55_ EAE [35]. Recombinant MOG (rMOG) is also popular to induce EAE, and B cells are fully activated and play an important role in the rMOG EAE [32].

## Materials and methods

### Animals

Female C57BL/6 mice weighing 14–16 g were purchased from Vital River Laboratory Animal (Beijing, P. R. China) and maintained at Harbin Medical University under specific-pathogen-free conditions at 18–29 °C and 40–70% humidity. All animal handling and experimental procedures were performed in accordance with the guidelines of the Care and Use of Laboratory Animals published by the China National Institute of Health.

### EAE induction

Mice were immunized subcutaneously with 100 μg recombination MOG (rMOG, GQFRVIGPGYPIRALVGDEAELPCRISPGKNATGMEVGWYRSPFSRVVHLYRNGKDQDAEQAPEYRGRTELLKETISEGKVTLRIQNVRFSDEGGYTCFFRDHS YQEEAAMELKVED) peptide or 200 μg MOG_35–55_ (MEVGWYRSPFSRVVHLYRNGK) peptide emulsified in complete Freund’s adjuvant (Sigma, St. Louis, MO, USA) containing *Mycobacterium tuberculosis* H37Ra (Difco Laboratories, Detroit, MI, USA) on 0 day and then were injected intravenously with 300 ng pertussis toxin (PT, LIST BIOLOGICAL LABORATORIES, INC.) both immediately after immunization and 2 days later. Clinical score was assessed daily according to the following scoring criteria: 0, no detectable signs of EAE; 1, limp tail; 2, hind limb weakness or impaired gait; 3, complete hind limb paralysis; 4, paralysis of fore and hind limbs; and 5, moribund or death. 0.5 was added to the lower score when clinical signs were intermediate between two grades of disease.

### BMSC cell culture and supernatant collection

The bone marrow stromal stem cells of mouse origin were kindly provided by Stem Cell Bank, Chinese Academy of Sciences. A single-cell suspension was made with BMSC culture media with 10% FBS and was plated at a density of 1 × 10^5^/cm^2^ in T-25 flanks and incubated at 37 °C in 5% CO_2_. Non-adherent cells were removed after 24 h; the medium was changed every 3 days until the colonies reached 70–80% confluence. Passage 9–11 cells were harvested and centrifuged at 300×*g* for 10 min for the evaluation of surface marker expression; the culture supernatant of BMSC were also collected. The supernatant collected from the different batches were uniformly mixed and stored separately for subsequent experiments. Related markers (CD29, CD31, CD34, CD44, CD90.2, CD117, Sca-1) of BMSC stained by flow cytometry are shown in Additional file 1: Figure S1.

### Intranasal administration

The mice were anesthetized with isoflurane to a shallow coma state. The mice were held at 45° by one hand, and the pipette was slowly dropped into the BMSC supernatant. Culture medium was used as a control group: from the third day after immunization until the onset of clinical symptoms, 60 μl per mouse (30 μl on each nostril) per day.

### Histological analysis

Mice of the control group and BMSC supernatant group at the peak stage of EAE were anesthetized and euthanized with pentobarbital and transcardially perfused with saline to eliminate the blood and then with buffered 4% paraformaldehyde. Spinal cords were removed and fixed in 4% paraformaldehyde. Paraffin-embedded 4-μm-thick spinal cord cross sections were stained with Luxol fast blue (LFB) for examination of demyelination. After being transcardially perfused, immediately remove and snap freeze fresh brain tissue in liquid nitrogen and keep at − 70 °C. Embed the tissue completely in OCT compound prior to frozen section. Cut the sections at 8-μm-thick, and after circling with PAP pen, the sections were fixed with cold acetone for 15 min at RT. For immunohistochemical studies, the sections were rinsed well three times in Tris-buffered saline with 0.5% Tween for 5 min, incubated in hydrogen peroxide, and then rinsed three times as above. Sections were incubated overnight at 4 °C with the primary antibodies. The sections were then rinsed well and incubated for 1.5 h at RT with appropriate horseradish peroxidase secondary antibodies for the DAB color development method. Antibodies used in the study are rat-anti-mouse CD45R (1:200), rat-anti-mouse CD4 (1:200), goat-anti-mouse Iba-1 (1:100), rat-anti-mouse CD68 (1:100), rat-anti-mouse CD86 (1:200), and rat-anti-mouse P2ry12 (1:50). And secondary antibodies used in the study include horseradish peroxidase-conjugated AffiniPure rabbit-anti-rat IgM (1:200) and horseradish peroxidase-conjugated AffiniPure donkey-anti-goat IgM (1:200).

Demyelination and immunopositively infiltrating cells were determined using an Olympus microscope (Olympus BX51). Image analyses were conducted using ImageJ. The number of demyelination per spinal cord cross section was counted manually from *n* = 3 sections per spinal cord from 3 mice in the peak stage (clinical score ≥ 2.5). The number of infiltrating cells per brain cross section was counted manually from sections per brain from 3 mice in peak stage (clinical score ≥ 2.5). Raw immunohistochemical micrographs were taken under the same conditions within pre-defined regions of interest and *n* ≥ 3 sections per brain.

### Preparation of mononuclear cells

Mice were anesthetized with pentobarbital and euthanized. The spleen of EAE and CFA mice were removed on the peak timing of EAE, minced into single-cell suspensions, then filtered through a 40-μm cell strainer (BD Biosciences, San Jose, CA, USA). Subsequently, mononuclear cells were suspended in PBS for further analysis.

### Cell purification and sorting

B cells isolated from the spleen of EAE and CFA mice were purified using the MojoSort™ Mouse CD19 Nanobeads (Biolegend, San Diego, CA) according to manufacturer’s instruction. T cells isolated from the spleen of EAE and CFA mice were purified using the MojoSort™ Mouse CD4 Nanobeads (Biolegend, San Diego, CA) according to the manufacturer’s instruction.

### In vitro cell culture

Splenic lymphocytes were cultured in vitro for 96 h in 1640 medium containing 10% fetal bovine serum after sorting or without sorting, during which rMOG was used for culture at 10 μg/ml, and culture in 6-well plate, 6 ml per well, cell density of 3 million/ml. One hundred microliters of BMSC supernatant was added into 900-μl medium (BMSC supernatant:total medium = 1:10). The ratio of T and B cells in co-culture assays, the ratio of T:B = 1:2, is similar to the ratio in the vivo.

### Flow cytometry and analysis

Single-cell lymphocyte suspensions were prepared, as described above. For intracellular cytokine staining, mononuclear cells were stimulated for 4 h with GolgiStop protein transport inhibitor containing monensin (BD Biosciences, San Jose, CA, USA) before staining. Next, single, resuspended cells were prepared in wash buffer containing PBS with 0.1% sodium azide. Antibodies specific to the respective cell-surface markers were diluted in appropriate volumes of staining buffer (BD Biosciences, San Jose, CA, USA) containing 1% BSA and incubated with cells for 30 min at 4 °C. After cells were washed twice with staining buffer, cells were fixed and permeabilized with fixation/permeabilization solution (BD Biosciences, San Jose, CA, USA) for 30 min at 4 °C. Antibodies specific to intracellular markers were diluted to the appropriate volume in perm/wash buffer (BD Biosciences, San Jose, CA, USA). Incubations were performed in the dark, and flow cytometric data were acquired using a FACSCalibur and FACS Verse flow cytometer (BD Biosciences, San Jose, CA, USA) and analyzed by FlowJo software (Treestar, Ashland, OR, USA). The antibodies used in the flow cytometry are listed in (Additional file 2: Table S1).

### ELISA

Quantitative analysis of IFN-γ, GM-CSF, IL-1β, and TNF-α levels and antibody levels were performed by ELISA using commercially available kits (Peprotech, USA). Measurements were made on serum samples from the two groups of mice, as well as on the fresh supernatant derived from 4-day culture of mononuclear cells stimulated by rMOG peptide (10 mg/mL) alone or with the presence of BMSCs.

### Statistical analysis

All statistical analysis was performed using GraphPad Prism (GraphPad Software Inc., La Jolla, CA). Statistical analyses included comparisons with the *T* test, two-way ANOVA, as appropriate; *P* value less than 0.05 was considered statistically significant.

## Results

### BMSC supernatant ameliorated EAE clinical course

**Fig. 1:**
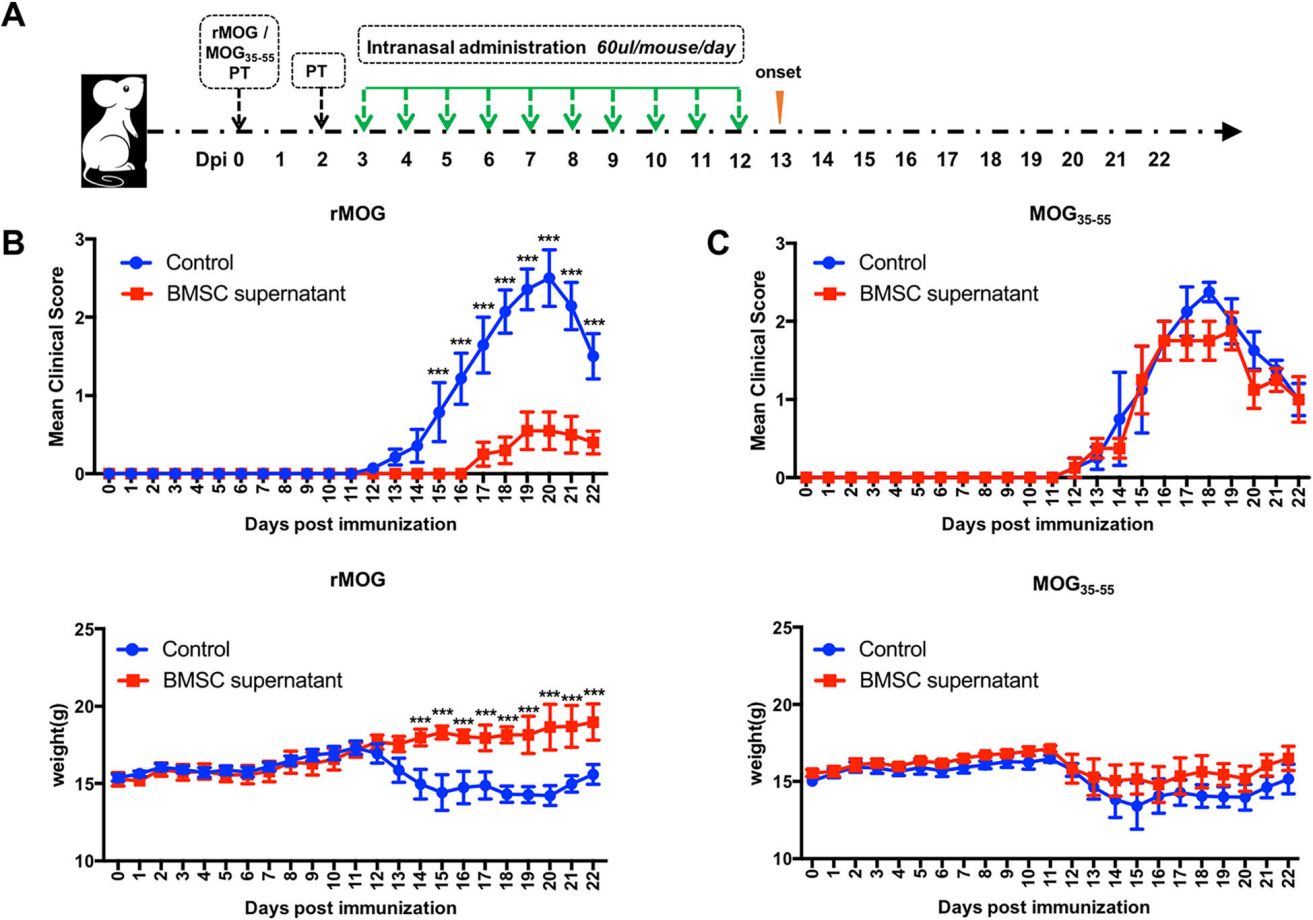
BMSC supernatant ameliorated EAE clinical course. **a** The EAE model was established in C57BL/6 mice, intranasally administered with BMSC supernatant (BMSC supernatant group) or medium control (control group) from day 3 post immunization until clinical symptom appeared. **b** EAE clinical course (upper panel) and body weight (lower panel) of rMOG-induced EAE, mean ± SEM, *n* = 14, ***P < 0.001. **c** EAE clinical course (upper panel) and body weight (lower panel) of MOG_35–55_-induced EAE, mean ± SEM, *n* = 6

MS has traditionally been regarded as an autoimmune disease mediated by T cells, but therapeutic effects on T cells have not achieved ideal results. The role of B cells in multiple sclerosis has been of increasing concern. Therefore, we constructed T cell-dominated (MOG_35–55_ induced) and B cell-dominated (rMOG induced) EAE models, respectively. In the basis of the successful construction of the EAE model, BMSC supernatant were administered to the mice via intranasal administration (Fig. 1a) and the clinical scores and body weights were recorded. We found that after intranasal administration of BMSC supernatant, the day when the EAE onset was significantly delayed, the disease course was significantly less severe compared with the control group (Fig. 1b), and the incidence was reduced (Additional file 3: Table S2). In the EAE model induced by MOG_35–55_, there were no significant effects of BMSC supernatant treatment (Fig. 1c).

### Effects of BMSC supernatant treatment on demyelination of the spinal cord and central nervous system

**Fig. 2:**
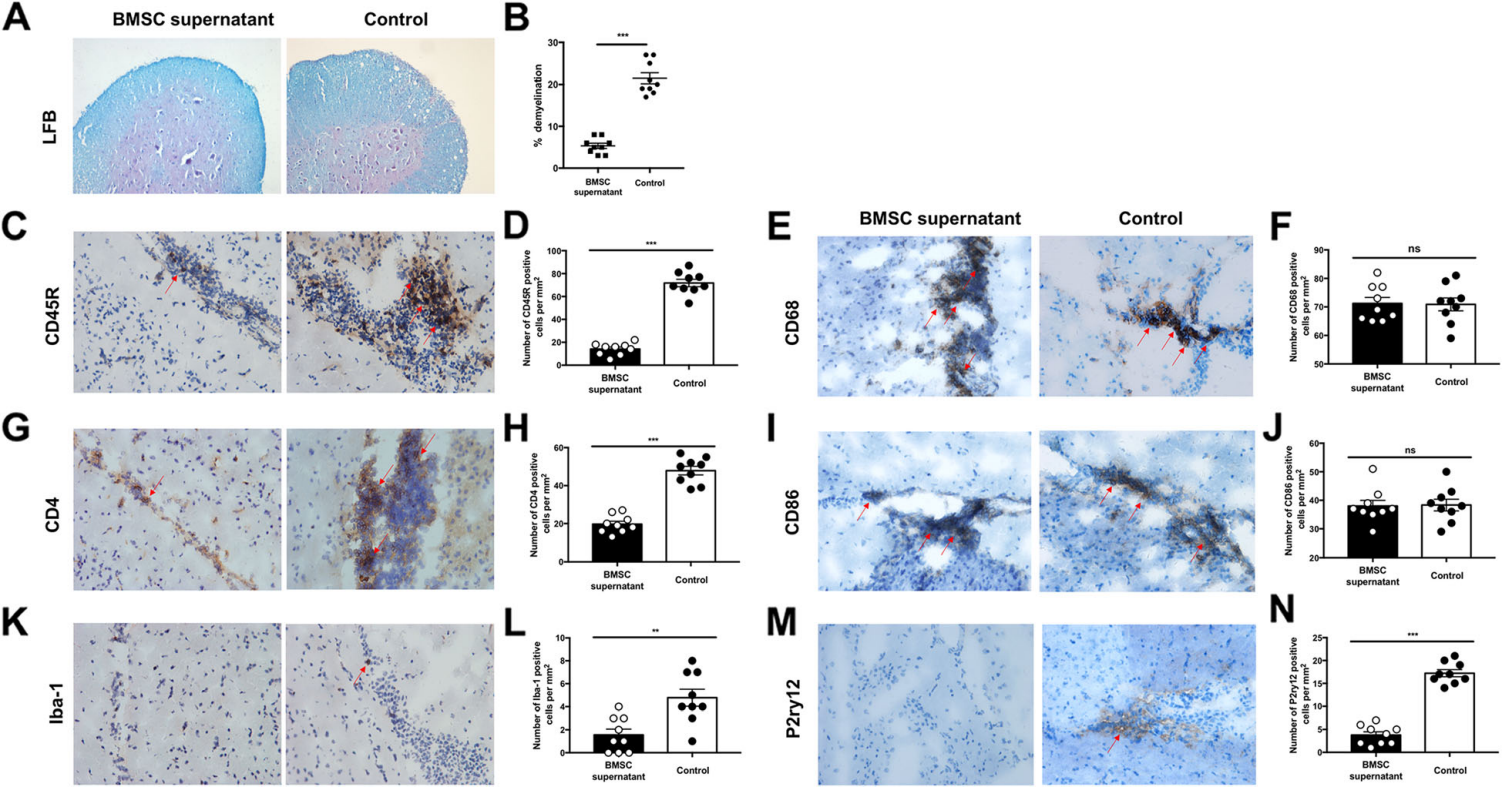
Effects of BMSC supernatant treatment on demyelination of the spinal cord and brain. **a** Demyelination for lumbar spinal cord sections at peak phase by Luxol fast blue staining, magnification × 100. In brain sections, cell infiltration was observed in the injured area of EAE mice (indicate by red arrow), **c** CD45R-positive B cells, **e** CD68-positive mononuclear cells, **g** CD4-positive T cells, **i** CD86-positive antigen presenting cells, and **k** Iba-1-positive and **m** P2ry12-positive microglia, magnification × 200. Percentage area of demyelination in the spinal cord white matter (**b**) and the number of positive cells per mm^2^ (**d**, **f**, **h**, **j, l, n**) calculated by analysis of transverse sections at three separate levels per mouse. Data represent the mean ± SEM of three individual mice per group, **P < 0.01, ***P < 0.001

The demyelination of the spinal cord and the inflammatory infiltration of the CNS can reflect the severity of EAE. We took the rMOG-immunized mice at the peak phase; on the 19th day after immunization, the spinal cord was stained with LFB. Compared with the control group, the degree of demyelination was significantly reduced in the experimental group (Fig. 2a, b). At the same time, we performed immunohistochemical staining of the brain tissue of the peak phase, and we found that the infiltration of B cells (CD45R positive, Fig. 2c, d) and T cells (CD4 positive, Fig. 2g, h) in the brain tissue of the experimental group was significantly reduced, and activation of microglia was decreased (Iba-1 positive, Fig. 2k, l, and P2ry12 positive, Fig. 2m, n). However, BMSC supernatant treatment had no significant effect on the number of monocytes infiltrating the central nervous system (CD68 positive, Fig. 2e, f) and had no significant effect on antigen-presenting cells (CD86 positive, Fig. 2i, j).

### BMSC supernatant treatment reduced antibody titer and inflammatory factor expression levels in the peripheral blood

**Fig. 3:**
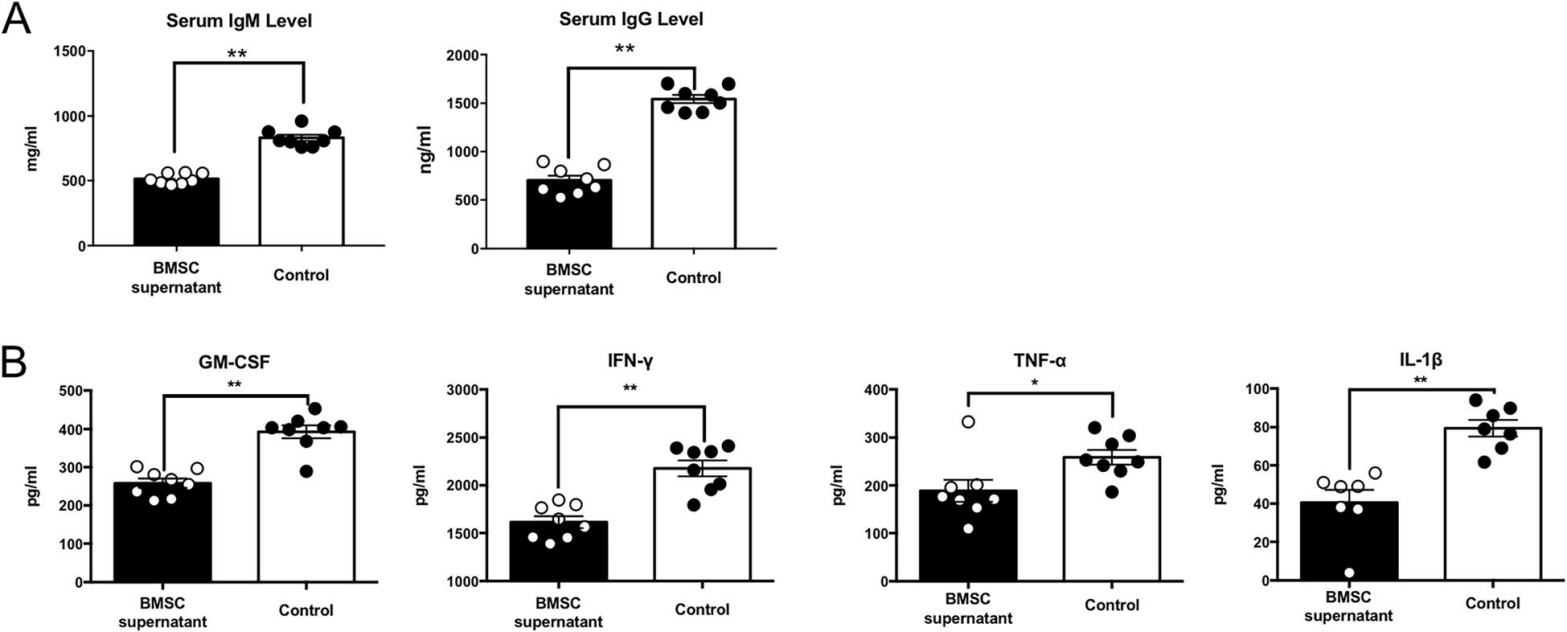
BMSC supernatant treatment reduced antibody titer and inflammatory factor expression levels in the peripheral blood. **a** IgM and IgG levels in peripheral blood serum on day 19 after immunization (data represents IgM or IgG levels for all eight mice in each group; error bars are SEM; **P < 0.01). **b** GM-CSF, IFN-γ, TNF-α, and IL-1β secretion in peripheral blood serum on day 19 after immunization (data represents serum IgM or IgG levels for all eight mice in each group; error bars are SEM; *P < 0.05, **P < 0.01)

As we mentioned earlier, the role of B cells in MS has received increasing attention, and B cells may participate in multiple sclerosis through multiple routes. As one of the most classic functions of B cells, we examined the levels of IgM and IgG in serum and found that the expression of IgM and IgG decreased after BMSC supernatant treatment (Fig. 3a). Next, we examined the expression of inflammatory factors in peripheral blood of mice at the peak stage and found that the pro-inflammatory factors GM-CSF, IL-1β, TNF-α, and IFN-γ were reduced after BMSC supernatant treatment (Fig. 3b).

### Effect of BMSC supernatant treatment on B cell function

**Fig. 4:**
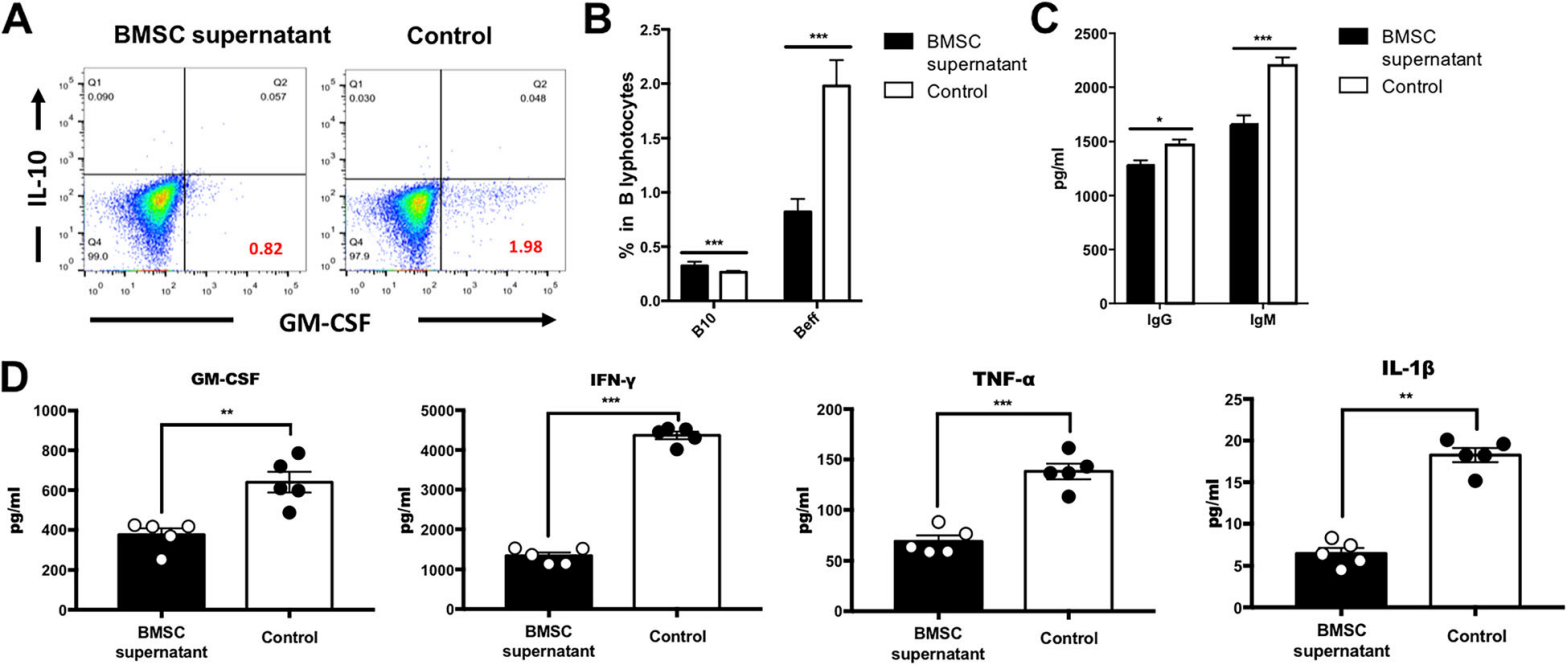
Effect of BMSC supernatant treatment on B cell function. We took B lymphocytes (CD19^+^) from the spleen at the peak phase of EAE mice and cultured them in vitro with BMSC supernatant or stem cell-specific medium as control. **a** Proportion of GM-CSF producing B cells and IL-10 producing B cells (gated in CD19^+^ B cells) after in vitro treatment of BMSC supernatant. **b** Statistical analysis of the ratio of GM-CSF producing B cells and IL-10 producing B cells after in vitro treatment of BMSC supernatant, mean ± SEM, *n* = 5, ***P < 0.001. **c** After BMSC supernatant treatment, the cell culture supernatant was collected, and the secretion of antibodies by B cells in the cell culture supernatant was detected by ELISA, mean ± SEM, *n* = 5, *P < 0.05, ***P < 0.001. **d** Secretion of cytokines GM-CSF, IFN-γ, TNF-α, and IL-1β of B cells after BMSC supernatant treatment, mean ± SEM, *n* = 5, *P < 0.05, ***P < 0.001

To explore how BMSC supernatant work in EAE, we did the following in vitro experiments. As we had obtained previously, BMSC supernatant have significant therapeutic effects on the EAE model of rMOG immunization, the B cell-mediated EAE model. B cells are one of the crucial factors for MS confirmed in recent years, B lymphocytes from the spleen at peak phase of rMOG-immunized EAE model were cultured in vitro, and BMSC supernatant were treated at the same time for 96 h. We found that the proportion of B lymphocytes secreting GM-CSF (effective B cell, Beff) in BMSC supernatant-treated group was significantly reduced compared with that in the control group as detected by flow cytometry (Fig. 4a, b), reflecting the anti-inflammatory effect of BMSC supernatant. In addition, we detected antibody secreted by B lymphocytes and found that IgM and IgG secretions were significantly reduced after BMSC supernatant treatment (Fig. 4c), and the secretions of pro-inflammatory factors GM-CSF, IFN-γ, TNF-α, and IL-1β were significantly reduced in the culture supernatant (Fig. 4d).

### B lymphocytes play a role by affecting T lymphocytes

**Fig. 5:**
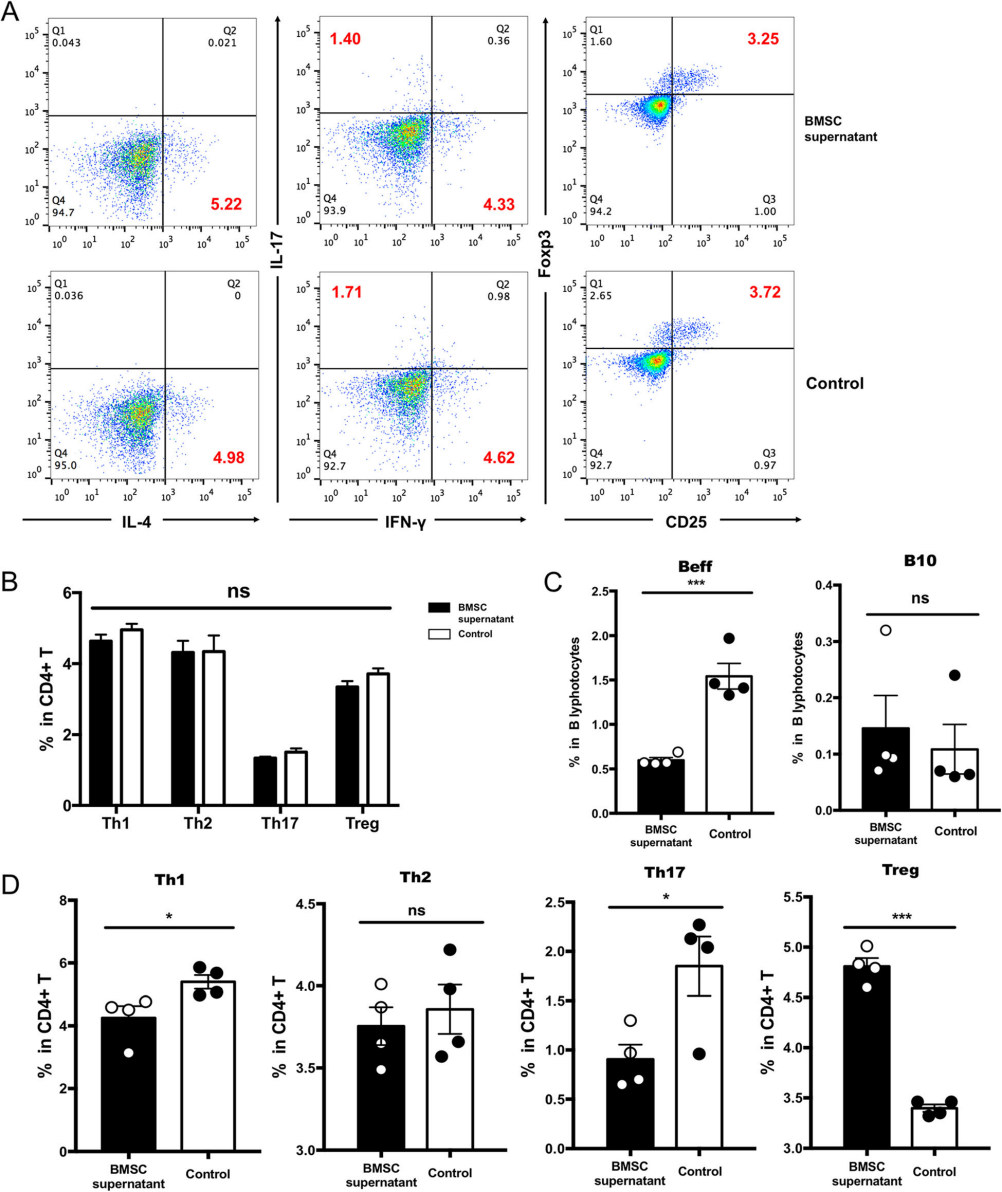
B lymphocytes play a role by affecting T lymphocytes. **a** Peripheral lymphoid tissues at the peak phase of EAE mice were selected. After positively sorting B cells, the remaining cells, T cells and other immune cell components, were cultured in vitro. Four subtypes of T cells were detected after in vitro treatment of BMSC supernatant. **b** Statistical analysis of the four subtypes of T cells after in vitro treatment of BMSC supernatant, mean ± SEM, *n* = 4. **c** We separately sorted the T cells (CD4^+^) and B cells (CD19^+^) in the peripheral immune tissues of the EAE model mice and co-cultured them in vitro. Changes in the proportion of B cells co-cultured after in vitro treatment of BMSC supernatant, mean ± SEM, *n* = 4, ***P < 0.001. **d** Changes in the proportion of T cells co-cultured after in vitro treatment of BMSC supernatant, mean ± SEM, *n* = 4, *P < 0.05, ***P < 0.001

As one of the typical pathogenesis of EAE, the proportion of pro-inflammatory/regulatory T cell subtypes plays an undeniable role in EAE. We next examined the proportion of subtypes of T cells treated with BMSC supernatant in vitro. T lymphocytes with other cells (after positive selected, removing CD19^+^ B cells) from the spleen at peak phase of rMOG-immunized EAE model were cultured in vitro, and BMSC supernatant were treated at the same time for 96 h; flow cytometry data showed there were no changes in the proportion of pro-inflammatory/regulatory T cell subtypes (Fig. 5a, b). Consistent with the results ex vivo, BMSC supernatant had no effect on the proportion of pro-inflammatory/regulatory T cell subtypes without B cells. Next, we co-cultured the sorted T cells and B cells from the spleen, draining lymph nodes by rMOG-immunized EAE model, and treated with BMSC supernatant for 96 h. Flow cytometry revealed that the proportion of GM-CSF-producing, pro-inflammatory B cells (Beff) was decreased (Fig. 5c), while the proportion of pro-inflammatory Th1 and Th17 cells was reduced and the proportion of Treg cells was increased (Fig. 5d). In the absence of B cells (positive selection of B cells), there was no statistical difference in the proportion of pro-inflammatory T cells/regulatory T cells, although other cellular components were present at this time. However, when the cell components other than T cells and B cells are removed, the BMSC supernatant treatment can reduce the proportion of pro-inflammatory B cells, decrease the proportion of pro-inflammatory T cells, and increase the proportion of regulatory T cells. These demonstrated that the supernatant of BMSC could directly affect B cells and also influence the function of T cells in EAE through B cells.

### No differences among different routes of BMSC supernatant administration

**Fig. 6:**
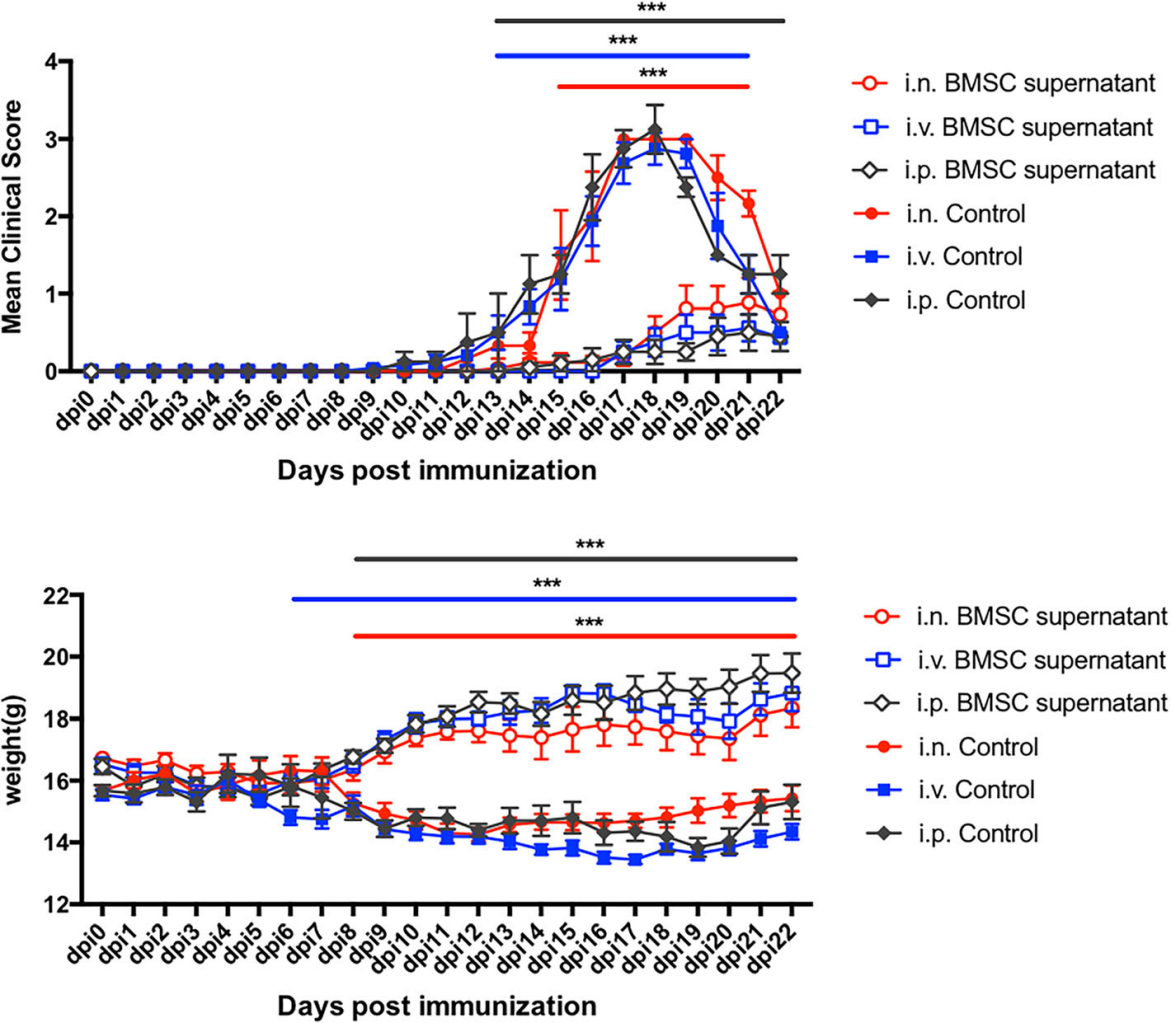
No differences among different routes of BMSC supernatant administration. On the third day after immunization, BMSC supernatant was treated by nasal mucosa, tail vein injection, and intraperitoneal injection. The nasal mucosa was unilaterally 30 μl per day, bilaterally administered; the intraperitoneal and tail vein doses were 60 μl per day, and the clinical score and body weight were terminated after the administration to the control group. Mean ± SEM, *n* = 8, ***P < 0.001

As mentioned earlier, the manner in which BMSC supernatant was administered through the nasal administration had significant therapeutic effects on EAE. We also compared the differences between this method and the therapeutic effects of the conventional treatment routes. Comparing the clinical scores and body weight changes of several routes of administration (Fig. 6), we could see that the therapeutic effects of nasal administration had no difference from the conventional route compared with the conventional administration route (nasal administration, i.n.), intravenous injection (i.v.), and intraperitoneal injection (i.p.), all had remarkable therapeutic effects.

## Discussion

The most striking aspect of this study was the use of BMSC supernatant for the beneficial effects of EAE by nasal administration. After the establishment of the EAE model, the clinical symptoms of EAE were significantly alleviated by continuous nasal administration of BMSC supernatant. The degree of demyelination in the central nervous system was reduced, inflammatory cell infiltration was decreased, microglia activation ratio was reduced, and secretion of inflammatory factors in peripheral blood was decreased.

In this experiment, we used the B cell-mediated EAE model. In previous studies, T cells were considered to be the main participants in EAE, but the efficacy of T cell-targeted therapy was not satisfactory and B cell therapy was achieved. The results suggest that the important role that B cells play in EAE is something we cannot ignore. We used the EAE model of rMOG immunization as the research object. It was confirmed in vitro that BMSC supernatant had a direct effect on B220^+^ B cells in vitro and had no direct effect on CD4^+^ T cells. In 2015, Li et al. reported that B cells secreting GM-CSF increased in proportion in patients with multiple sclerosis, and mononuclear/macrophage immunity was enhanced by GM-CSF, which promoted differentiation of pro-inflammatory Th cells [37]. The proportion of B cells secreting GM-CSF after treatment of BMSC supernatant was decreased, which changed the balance between pro-inflammatory B cells and regulatory B cells. In addition, we also confirmed that BMSC supernatant can affect CD4^+^ T cells through B220^+^ B cells; it can inhibit the differentiation of T cells into pro-inflammatory cytokines in EAE and reduce the secretion of pro-inflammatory factors. While these phenomena did not occur when treated with T cells alone; the above confirmed that BMSC supernatant can directly alter B cell function and can affect T cells through B cells. It can be seen that in EAE, B cells have direct pathogenic effects and can exert regulatory functions.

Previous studies have shown that BMSC may be a viable option for the treatment of CNS diseases. Transplanted BMSCs protect and repair damaged brains through various mechanisms [38, 39] or reduce inflammatory cell infiltration in the central nervous system [40, 41]. Ivasaki and colleagues report that intranasal BMSCs can migrate to the injured area and improve cognitive function in neonates after hypoxia-ischemia [42]. The olfactory nerve cells connect the nasal mucosa to the olfactory bulb and the frontal cortex of the brain. The peripheral trigeminal nerve is also directly connected to the nasal passages and the brainstem and spinal cord. This anatomical feature plays a crucial role in stem cell migration. Inflammatory factors, combined with dead blood cell debris, iron, thrombin, other chemokines, cytokines, and proteases, create a harsh environment for transplanted cells [43]. The use of BMSC cell therapy or recently reported treatment with BMSC exosomes [40, 44] and the treatment of BMSC supernatant by nasal mucosa in our study can all ameliorate the symptoms of EAE to some extent. For the mouse EAE model, BMSC supernatant nasal therapy and traditional treatment can improve the central nervous system demyelination and reduce the central nervous system inflammatory infiltration. Although the mechanism of BMSC treatment of EAE is not fully understood [45, 46], while BMSCs have been used for a long time in clinical practice, BMSC treatments have many side effects, like nausea [47], vomiting [48], infection [49, 50], and hematoma [51], Graft-versus-host disease [24, 52] and these side effects are uncontrollable. We administered BMSC supernatant through the nasal cavity to minimize the side effects of stem cell therapy, and it has obvious therapeutic effects. Through the comparison of the therapeutic effects of the nasal cavity and the conventional route, it is found that there is no difference between the nasal cavity and the conventional route; as a non-invasive and safe way, it provides a new idea for future clinical treatment.

## Conclusion

In conclusion, this study demonstrates that BMSC supernatant have a therapeutic effect on EAE. BMSC supernatant can improve the clinical symptoms of EAE, decrease the inflammatory changes of the central nervous system and demyelination, and reduce the secretion of inflammatory cytokines in peripheral blood. The therapeutic effect is to reduce the proportion of pro-inflammatory B cells, lessen the secretion of antibody titers by B cells, and reduce the secretion of inflammatory cytokines by B cells, thereby affecting the proportion of pro-inflammatory/inflammatory T cells and achieving therapeutic effects on EAE. However, detailed mechanisms need to be studied in subsequent experiments. This study provides evidence that the BMSC supernatant have a good therapeutic effect on EAE by nasal administration, which may provide a new potential therapeutic strategy for EAE treatment.

## Supplementary information

**Additional file 1: Figure S1.** Immunophenotypes of BMSCs after culture. BMSCs (passage 9–11) were cultured by stem cell-specific medium, and cell culture supernatant were collected. After that flow cytometry was used to detect BMSC-related markers, the blue lines represent labeled cells and the red lines represent negative control.

**Additional file 2: Table S1.** Antibody used in the flow cytometry.

**Additional file 3: Table S2.** BMSC supernatant Ameliorated EAE Clinical Course.

## Data Availability

All data and materials are available in the manuscript.

## Abbreviations

MS: Multiple sclerosis
CNS: Central nervous system
BMSCs: Bone marrow mesenchymal stem cells
EAE: Experimental autoimmune encephalomyelitis
rMOG: Recombination MOG
PT: Pertussis toxin
H & E: Hematoxylin and eosin
LFB: Luxol fast blue
GM-CSF: Granulocyte-macrophage colony-stimulating factor
Beff: Effective B cell
IL-1β: Interleukin *1β*
TNF-α: Tumor necrosis factor α
IFN- γ: Interferon-γ
IgM: Immunoglobulin M
IgG: Immunoglobulin G
IL-4: Interleukin-4
IL-17: Interleukin-17
Th1: T helper cell 1
Th17: T helper cell 17
Treg: Regulatory T cell
i.n.: Administration route
i.v.: Intravenous injection
i.p.: Intraperitoneal injection
