# Supplementary figures and images for: Treatment of the bone marrow stromal stem cell supernatant by nasal administration—a new approach to EAE therapy
Source: Stem Cell Res Ther. 2019 Nov 15;10:325. doi: 10.1186/s13287-019-1423-6 (PMC6858701; doi:10.1186/s13287-019-1423-6)

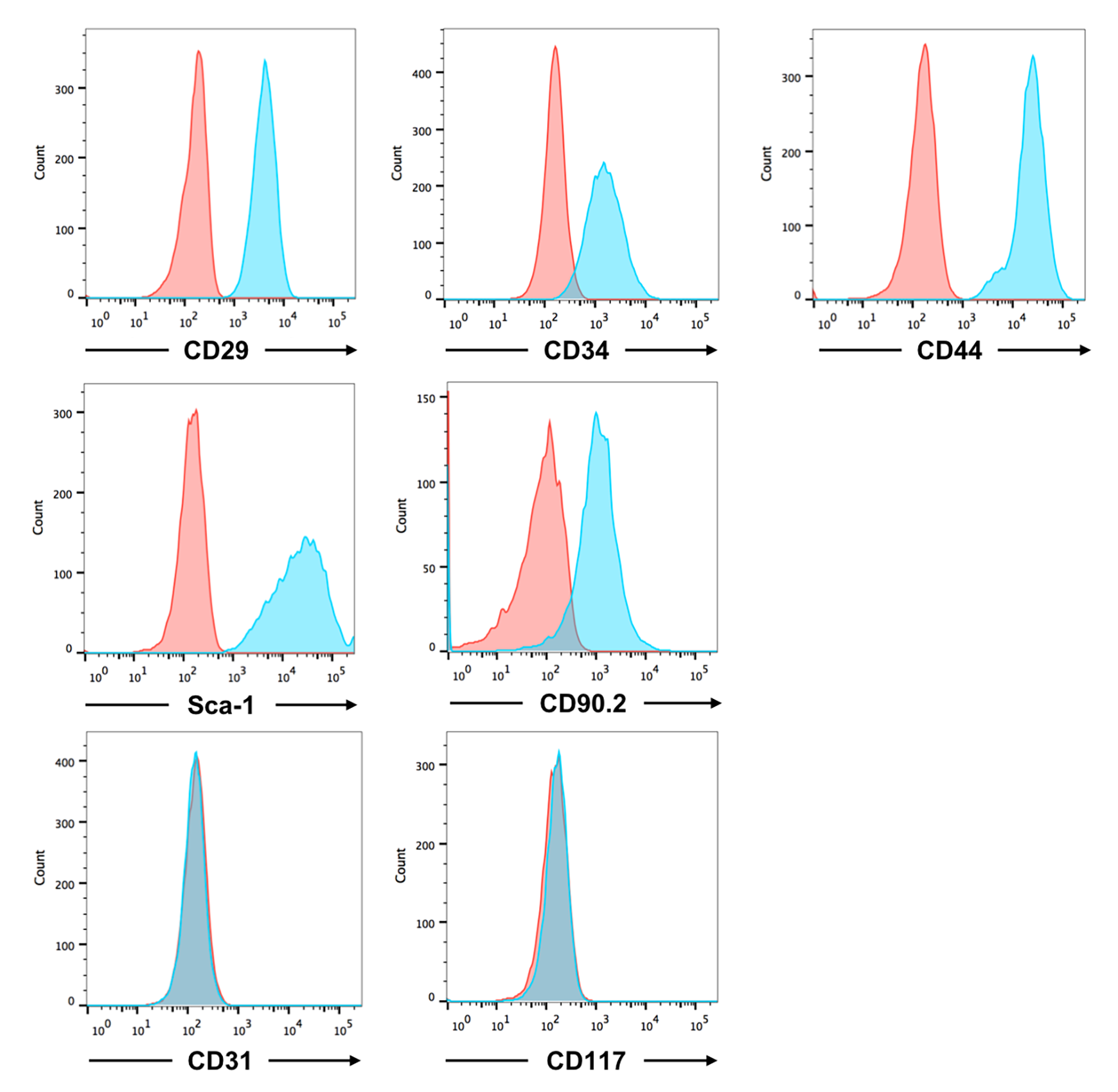

Supplement: Supplementary file 1 — Additional file 1: Figure S1. Immunophenotypes of BMSCs after culture. BMSCs (passage 9–11) were cultured by stem cell-specific medium, and cell culture supernatant were collected. After that flow cytometry was used to detect BMSC-related markers, the blue lines represent labeled cells and the red lines represent negative control. [file 13287_2019_1423_MOESM1_ESM.tif]
